# New insights on serodiagnosis of trichinellosis during window period: early diagnostic antigens from *Trichinella spiralis* intestinal worms

**DOI:** 10.1186/s40249-017-0252-z

**Published:** 2017-02-20

**Authors:** Zhong-Quan Wang, Ya-Li Shi, Rou-Dan Liu, Peng Jiang, Ya-Yi Guan, Ying-Dan Chen, Jing Cui

**Affiliations:** 1 Department of Parasitology, Medical College, Zhengzhou University, Zhengzhou, 450052 China; 2 National Institute of Parasitic Diseases, Chinese Center for Disease Control and Prevention, Key Laboratory of Parasite and Vector Biology, Ministry of Health, Shanghai, 200025 China

**Keywords:** Trichinellosis, Early serodiagnosis, Intestinal infective larvae, Adult worms

## Abstract

The clinical diagnosis of trichinellosis is difficult because its clinical manifestations are nonspecific. Detection of anti-*Trichinella* IgG by ELISA using *T. spiralis* muscle larval excretory-secretory (ES) antigens is the most commonly used serological method for diagnosis of trichinellosis, but the main disadvantage is false negativity during the early stage of infection. There is an obvious window period between *Trichinella* infection and antibody positivity.

During the intestinal stage of *Trichinella* infection, the ES antigens of intestinal worms (intestinal infective larvae and adults) are exposed to host’s immune system at the earliest time and elicit the production of specific anti-*Trichinella* antibodies. Anti-*Trichinella* IgG antibodies in infected mice were detectable by ELISA with ES antigens of intestinal worms as soon as 8–10 days post infection (dpi), but ELISA with muscle larval ES antigens did not permit detection of infected mice before 12 dpi. Therefore, the new early antigens from *T. spiralis* intestinal worms should be screened, identified and characterized for early serodiagnosis of trichinellosis.

**Electronic supplementary material:**

The online version of this article (doi:10.1186/s40249-017-0252-z) contains supplementary material, which is available to authorized users.

## Multilingual abstracts

Please see Additional file 1 for translations of the abstract into the six official working languages of the United Nations.

## Background

Trichinellosis is a major food-borne parasitic zoonosis acquired by eating raw or undercooked meat contaminated with the infective larvae of the genus *Trichinella*. Human trichinellosis has been documented in 55 countries in the world [1]. From 1986 to 2009, there were 65 818 cases and 42 deaths reported from 41 countries, and is considered to be a neglected infectious disease, especially in developing countries of east Europe, south America, Asia and Africa [2, 3]. From 2004 to 2009 in China, 15 outbreaks of human trichinellosis affected 1 387 people and caused 4 deaths. Pork is the predominant source of outbreaks of human trichinellosis in China. Out of 15 outbreaks, 12 (85.71%) were caused by eating raw or undercooked pork [4]. Swine trichinellosis is transmitted mostly through garbage (i.e., feeding pigs with raw swill). Pigs infected with *Trichinella* are predominately from small backyard farms where animals are raised under poor hygienic conditions, and from rural and mountainous areas where they range freely at pasture. Pigs were sometimes slaughtered at home in rural and mountainous areas without veterinary inspection [5, 6]. Recently, outbreaks of trichinellosis also occurred in the mountainous regions of Cambodia, Lao PDR, north Thailand and Vietnam, and most of the patients had consumed traditional raw or undercooked pork dishes at wedding, funeral, or New Year parties [7–10]. Trichinellosis has become an emerging and re-emerging zoonotic disease with health, social, and economic impacts in developing countries [11].

The clinical diagnosis of trichinellosis is difficult because its clinical manifestations are nonspecific. The definitive diagnosis of trichinellosis can be made by detecting larvae in a biopsy muscle samples or specific anti-*Trichinella* antibodies, but muscle biopsy is not sensitive to the light infections and the early stage of infection [12]. Serum specific anti-*Trichinella* IgE are thought to appear first and are typical of the acute stage of the disease, but IgE antibodies are detected in a few cases only at the onset of the disease because their half-life in serum is relative short [13]. The detection of anti-*Trichinella* IgG by ELISA using excretory–secretory (ES) antigens of *T. spiralis* muscle larvae (ML) is the most commonly used method for serodiagnosis of trichinellosis and is recommended by the International Commission on Trichinellosis (ICT), because ML are easily collected from experimentally infected animals and their ES antigens can be prepared by the *in vitro* cultivation of larvae [14]. However, the main disadvantage of the detection of anti-*Trichinella* IgG by using ML ES antigens is the occurrence of a high rate of false negative results during the early stage of infection [15], possibly because the majority of ML ES antigens are stage-specific and not recognized by specific antibodies induced by the parasites during the intestinal phase [16]. Previous studies have shown that 100% detection of anti-*Trichinella* IgG is not possible for at least 1–3 months after initial infection with the parasite [13]. There is an obvious window period of 3–4 weeks between *Trichinella* infection and specific antibody positivity. The indirect immunofluorescence test (IIF) with frozen sections of infected tissue or isolated whole ML as antigens could detect specific antibodies in sera of experimentally infected mice 1–2 weeks after infection or sera of patients with trichinellosis from day 6 after the onset of illness [17, 18], but cross-reactions with *Trichinella* antigens were observed in the patients with autoimmune diseases and other helminthiases because IIF is based on cuticle surface antigens of ML [19]. This is particularly important in regions where other human helminthiases (e.g., ascariasis, trichuriasis, clonorchiasis, paragonimiasis, cysticercosis and so on) are common and cross-reactions with these parasites could give could give false positive results [20]. Furthermore, persons reading the fluorescent sections should be aware that only sections with a uniform fluorescence should be considered as positive, whereas those, which show a non-uniform fluorescence along the cuticle, should be considered as false-positive.

*T. spiralis* circulating antigen (CAg) is the ES antigens produced by live worms and can directly enter the peripheral blood circulation, and CAg appears earlier than antibody in blood circulation [21]. Theoretically, the detection of *T. spiralis* CAg seems an early diagnostic method for trichinellosis. However, because the serum levels of *Trichinella* CAg are usually quite low, the detection rate of CAg in serum samples was usually only 30–50% in the patients with clinical trichinellosis [22]. Thus, detection of *T. spiralis* CAg has not been clinically applied to serodiagnosis of trichinellosis. So far, the detection of anti-*Trichinella* IgG is still the serological test of choice for diagnosis of trichinellosis. Hence, it is necessary to exploit the new source of early diagnostic antigens for detection of anti-*Trichinella* antibodies.

## Discussion

After ingestion, *T. spiralis* ML are released from their nurse-cells in the stomach and activated to intestinal infective larvae (IIL) by intestinal content or bile after 0.9 h post-infection (hpi). The IIL invade host’s intestinal epithelium, and undergo four molting to develop to adult worms (AW) in 31 hpi. After mating, the female AW invade intestinal mucosa again and live there for 10–20 days in mice and rats or 4–6 weeks in humans [23]. The IIL and AW are the first two invasive stages in life cycle of *T. spiralis*. During the intestinal stage of *T. spiralis* infection, the ES antigens produced by the IIL and AW result in early exposure to the host’s immune system and elicit the production of specific anti-*Trichinella* antibodies. The ES proteins of intestinal *T. spiralis* worms might contain the early diagnostic markers of trichinellosis. Therefore, the new early diagnostic antigens should be searched and identified from *T. spiralis* intestinal worms instead of the conventional ML.

Recent studies have showed that crude antigens of *T. spiralis* AW were recognized by sera from infected mice or swine 7 days post-infection (dpi) [24]. The recombinant protein from IIL at 6 hpi or pre-adults 20 hpi was recognized by pig antiserum in Western blot as early as 15–20 dpi [25, 26]. Anti-*Trichinella* IgG in mice infected with 100 *T. spiralis* larvae were detectable by ELISA with adult worm or IIL ES antigens as soon as 8 or 10 dpi, but ELISA with ML ES antigens did not permit detection of infected mice before 12 dpi. When the sera of patients with trichinellosis at 19 dpi were assayed, the sensitivity (100%, 20/20) of ELISA with adult or IIL ES antigens was evidently higher than 75% (15/20) of ELISA with ML ES antigens. The specificity of ELISA with AW (98.11%, 156/159) and IIL ES (96.86%, 154/159) antigens was also higher than 89.31% (142/159) of ELISA with muscle larval ES antigens. There were no cross-reactions of ELISA with AW and IIL ES antigens with sera of patients with schistosomiasis, clonorchiosis, sparganosis and healthy persons [27, 28]. The sensitivity and specificity of AW and IIL ES antigens were superior to those of muscle larval ES antigens. Therefore, the ES antigens of *T. spiralis* intestinal worms (IIL and AW) could be considered as a new source of diagnostic antigens and the potential antigens for the early specific serodiagnosis of trichinellosis.

However, there was also low cross-reactivity of the ELISA with the AW and IIL ES antigens with sera of a few of patients with paragonimiosis or cysticercosis. The *Trichinella-*specific protein bands in the AW and IIL ES antigens should be further identified and validated by Western blotting with a large-scale of sera of the patients with trichinellosis and other helminthiasis. Moreover, *Trichinella-*specific AW and IIL ES antigens recognized by early infection sera should be characterized by mass spectrometry [29, 30]. Then, the recombinant early antigens of *Trichinella* should be developed and their sensitivity and specificity were evaluated with a large-scale trial in future. The exploitation of the early diagnostic antigens from *T. spiralis* invasive stages may provide new insights into early serodiagnosis of other helminthiasis, for example, the early antigens from cercariae and schistosomula of *Schistosoma* for diagnosis of schistomiasis [31]. Additionally, it will also provide valuable information for further understanding the parasite-host interaction and invasion mechanism [32–34].

## Conclusions

The ES antigens of *T. spiralis* intestinal stages (intestinal infective larvae and adults) are exposed to host’s immune system at the earliest time and elicit the production of specific anti-*Trichinella* antibodies, the new antigens from *T. spiralis* intestinal worms should be screened, identified and characterized for early serodiagnosis of trichinellosis. The exploitation of the early diagnostic antigens from the parasite’s invasive stages may provide new insights into early serodiagnosis of other helminthiasis.

## Additional file

Additional file 1: Multilingual abstracts in the six official working languages of the United Nations. (PDF 698 kb)

## Abbreviations

CAg: Circulating antigen
dpi: days post-infection
ES: Excretory–secretory
hpi: hour post-infection
ICT: International Commission on Trichinellosis
IIL: Intestinal infective larvae
ML: Muscle larvae
PDR: People’s Democratic Republic.
