# Supplementary material for: New insights on serodiagnosis of trichinellosis during window period: early diagnostic antigens from Trichinella spiralis intestinal worms
Source: Infect Dis Poverty. 2017 Feb 20;6:41. doi: 10.1186/s40249-017-0252-z (PMC5319148; doi:10.1186/s40249-017-0252-z)
Supplement: Additional file 1: — Multilingual abstracts in the six official working languages of the United Nations. (PDF 698 kb) [file 40249_2017_252_MOESM1_ESM.pdf]

Translation of the abstract into the six official working languages of the United Nations

رؤى جديدة في التشخيص المصلي للخيطيات خلال فترة التي تفصل بين بداية حدوث الإصابة وتشخيصها الفعلي: مستضدات التشخيص في وقت مبكر للديدان الشعرينة الحلزونية المعوية

تشونج تشيوان وانج، يا لي شي، رو-دان ليو بنج جيانج، يا يي جوان، يينج دان وتشن جينج تسوى

ملخص

يصعب التشخيص السريري للخيطيات لأن المظاهر السريرية التي تشخصها غير محددة. يشيع الكشف عن الخيطيات بواسطة اختبار دراسة الجلوبلين المناعي IgG المضاد للشعرينة الحلزونية باستخدام مستضدات الديدان الشعرينة الحلزونية اليرقية الإفراغية-الإفرازية في العضلات الذي يعتبر الأسلوب المصلي الأكثر شيوعا لتشخيص الخيطيات، ولكن العيب الرئيسي في هذا الاختبار هو الحصول على نتائج سلبية كاذبة خلال المرحلة المبكرة من العدوى. وهناك فترة تفصل بين بداية حدوث الإصابة وتشخيصها الفعلي واضحة بين عدوى الديدان الخيطية والتشخيص الإيجابي للأجسام المضادة.

خلال المرحلة المعوية للعدوى بالديدان الخيطية، تتعرض الديدان للمستضدات الإفراغية-الإفرازية من الديدان المعوية (يرقات وبالغة معدية معوية) إلى نظام مناعة المضيف في أقرب وقت وتحث على إنتاج أجسام مضادة محددة لمكافحة الخيطيات. كانت الأجسام المضادة لمكافحة الخيطيات قابلة للكشف في الفئران المصابة بواسطة اختبار المناعة المرتبط بالإنزيم مع المستضدات الإفراغية-الإفرازية للديدان المعوية فيما بين 8-10 أيام من الأيام اللاحقة للعدوى، لكن اختبار المناعة المرتبط بالإنزيم مستضدات الديدان الشعرينة الحلزونية اليرقية الإفراغية-الإفرازية في العضلات لم يسمح بالكشف عن المرض في الفئران المصابة قبل 12 يوم من الأيام اللاحقة للعدوى. لذلك، ينبغي فحص المستضدات المبكرة الجديدة المأخوذة من الديدان المعوية الحلزونية، وتحديدها وتمييزها للتشخيص المصلي في وقت مبكر للخيطيات.

Translated from English version into Arabic by Mahmoud Sami, through

TRANSLATORS
WITHOUT BORDERS

旋毛虫病窗口期血清学诊断的新见解：旋毛虫肠道期虫体早期诊断抗原

王中全，师亚丽，刘若丹，姜鹏，官亚宜，陈颖丹，崔晶

摘要

由于旋毛虫病的临床表现是非特异性的，临床诊断较困难。应用旋毛虫肌幼虫排泄-分泌（ES）抗原ELISA检测抗旋毛虫抗体IgG，是目前诊断旋毛虫病最常用的血清学方法，但其主要缺点是在感染早期出现假阴性结果，在旋毛虫感染与抗体阳性之间具有明显的窗口期。

在旋毛虫感染的肠道期，肠道期虫体（肠道感染性幼虫和成虫）的 ES 抗原最早暴露于宿主的免疫系统，诱导宿主产生特异性的抗旋毛虫抗体。应用肠道期虫体 ES 抗原 ELISA，在小鼠感染旋毛虫后 8-10 d 即可检出抗旋毛虫抗体 IgG；但应用肌幼虫 ES 抗原 ELISA，在小鼠感染旋毛虫后 12 d 之前则不能检出抗旋毛虫抗体 IgG。因此，应从旋毛虫肠道期虫体筛选与鉴定新的早期诊断抗原，然后用于旋毛虫病的早期血清学诊断。

Translated from English version into Chinese by Zhong-Quan Wang, Jing Cui

Nouvelle percée dans le sérodiagnostic de la trichinose durant la période de latence sérologique : diagnostic précoce grâce aux antigènes de vers intestinaux *Trichinella spiralis*

Zhong-Quan Wang, Ya-Li Shi, Rou-Dan Liu, Peng Jiang, Ya-Yi Guan, Ying Dan Chen et Jing Cui

Résumé

Étant donné que les manifestations cliniques de la trichinose sont non spécifiques, le diagnostic clinique est difficile. La méthode de sérodiagnostic la plus utilisée est la détection des anticorps IgG anti-*Trichinella* par ELISA au moyen d'antigènes d'excrétion-sécrétion (ES) de larves musculaires de *T. spiralis*; or, son principal inconvénient est le risque de faux négatif en début d'infection. Il y a une période de latence sérologique évidente entre l'infection et le moment où les anticorps sont détectables.
Lors de la phase intestinale de l'infection à *Trichinella,* les antigènes ES des vers intestinaux (larves et vers adultes) sont les premiers à entrer en contact avec le système immunitaire de l'hôte et à susciter la production d'anticorps spécifiques contre *Trichinella.* Dans le cadre de cette étude, un test ELISA utilisant des antigènes ES de vers intestinaux a permis de détecter des anticorps IgG anti-*Trichinella* chez des souris infectées aussi tôt que 8 à 10 jours après l'infection. En revanche, cette détection n'était pas possible avant 12 jours par ELISA utilisant des antigènes ES de larves musculaires. Les nouveaux antigènes devraient donc être criblés, identifiés et caractérisés à des fins de sérodiagnostic précoce de la trichinose.

Translated from English version into French by Edgar Inc., through

Новый взгляд на серологическую диагностику трихинеллёза в период серонегативного окна: диагностический антиген на ранних стадиях, выделенный из кишечных червей *Trichinella spiralis*

Джонг-Кван Ванг/Zhong-Quan Wang, Йа-Ли Ши/Ya-Li Shi, Роу-Дан Лиу/Rou-Dan Liu, Пенг Джианг/Peng Jiang, Йа-Йи Гуан/Ya-Yi Guan, Йинг Дан Чен/Ying Dan Chen и Джинг Суи/Jing Cui

Краткое содержание

Клиническая диагностика трихинеллёза осложняется тем фактом, что клиническое проявление заболевания не является специфическим. Определение анти-*трихинеллёзного иммуноглобулина G* с помощью твердофазного иммуноферментного анализа (ELISA) с использованием *T.spiralis* мышечных ларвальных экскреторно-секреторных (ЭС) антигенов является наиболее распространенным серологическим методом диагностиком трихинеллёза, но основным недостатком является ложный отрицательный результат, получаемый на ранних стадиях развития инфекции. Имеется очевидный период серонегативного окна между развитием инфекции *Trichinella* и положительным результатом на антитела.
Во время кишечной стадии развития инфекции *Trichinella*, антитела ЭС кишечных червей (кишечные инвазионные личинки и взрослые особи) подвергаются воздействию со стороны иммунной системы носителя на ранних стадиях и вызывают выработку специфических анти-трихинеллезных антител. Антитела анти-*трихинеллёзного иммуноглобулина G* у инфицированных мышей можно было определить с помощью твердофазного иммуноферментного анализа с использованием ЭС антигенов кишечных червей через 8-10 дней после инфицирования (dpi), но твердофазный иммуноферментный анализ с использованием мышечных ларвальных ЭС антигенов не позволял определить антитела у инфицированных мышей до 12 дня после инфицирования. Следовательно, следует проводить скрининг новых антигенов на ранних стадиях, выделенных из кишечных червей *T. Spiralis*, а также следует провести их идентификацию и дать им характеристику для проведения серологической диагностики трихинеллёза на ранних стадиях.

Translated from English version into Russian by Helga_Sh, through

Nuevos conocimientos sobre el serodiagnóstico de la triquinosis durante el período silente: antígenos de diagnóstico temprano con parásitos intestinales *Trichinella spiralis*

Zhong-Quan Wang, Ya-Li Shi, Rou-Dan Liu, Peng Jiang, Ya-Yi Guan, Ying Dan Chen and Jing Cui

Resumen
El diagnóstico clínico de la triquinosis es difícil debido a que sus manifestaciones clínicas son inespecíficas. La detección de la IgG anti-*Trichinella* mediante un ELISA utilizando antígenos excretores-secretores (ES) larvales musculares de *T. spiralis* es el método serológico más comúnmente utilizado para el diagnóstico de la triquinosis, pero la principal desventaja es la falsa negatividad durante la etapa temprana de la infección. Existe un periodo silente evidente entre la infección por *Trichinella* y la positividad del anticuerpo.
Durante la fase intestinal de la infección por *Trichinella*, los antígenos ES de parásitos intestinales (larvas y adultos infecciosos intestinales) están expuestos al sistema inmune del huésped en el momento más temprano y provocan la producción de anticuerpos anti-*Trichinella* específicos. Los anticuerpos anti-*Trichinella* de la IgG en ratones infectados fueron detectables mediante un ELISA con antígenos ES de parásitos intestinales 8-10 días post-infección (dpi), pero un ELISA con antígenos ES larvales musculares no permitió la detección de ratones infectados antes de 12 dpi. Por lo tanto, los nuevos antígenos tempranos de los parásitos intestinales de *T. spiralis* deben ser examinados, identificados y caracterizados para el serodiagnóstico temprano de la triquinosis.

Translated from English version into Spanish by Amparo Muñoz, through
